# Systematic genotyping of groups of cows to improve genomic estimated breeding values of selection candidates

**DOI:** 10.1186/s12711-016-0250-9

**Published:** 2016-09-28

**Authors:** Laura Plieschke, Christian Edel, Eduardo C. G. Pimentel, Reiner Emmerling, Jörn Bennewitz, Kay-Uwe Götz

**Affiliations:** 1 Bavarian State Research Center for Agriculture, Institute of Animal Breeding, Prof.-Dürrwaechter-Platz 1, 85586 Poing-Grub, Germany; 2 Institute of Animal Science, University Hohenheim, Garbenstraße 17, 70599 Stuttgart, Germany

## Abstract

**Background:**

Extending the reference set for genomic predictions in dairy cattle by adding large numbers of cows with genotypes and phenotypes has been proposed as a means to increase reliability of selection decisions for candidates.

**Methods:**

In this study, we explored the potential of increasing the reliability of breeding values of young selection candidates by genotyping a fixed number of first-crop daughters of each sire from one or two generations in a balanced and regular system of genotyping. Using stochastic simulation, we developed a basic population scenario that mimics the situation in dual-purpose Fleckvieh cattle with respect to important key parameters. Starting with a reference set consisting of only genotyped bulls, we extended this reference set by including increasing numbers of daughter genotypes and phenotypes. We studied the effects on model-derived reliabilities, validation reliabilities and unbiasedness of predicted values for selection candidates. We also illustrate and discuss the effects of a selected sample and an unbalanced sampling of daughters. Furthermore, we quantified the role of selection with respect to the influence on validation reliabilities and contrasted these to model-derived reliabilities.

**Results:**

In the most extended design, with 200 daughters per sire genotyped from two generations, single nucleotide polymorphism (SNP) effects were estimated from a reference set of 420,000 cows and 4200 bulls. For this design, the validation reliabilities for candidates reached 80 % or more, thereby exceeding the reliabilities that were achieved in traditional progeny-testing designs for a trait with moderate to high heritability. We demonstrate that even a moderate number of 25 genotyped daughters per sire will lead to considerable improvement in the reliability of predicted breeding values for selection candidates. Our results illustrate that the strategy applied to sample females for genotyping has a large impact on the benefits that can be achieved.

**Electronic supplementary material:**

The online version of this article (doi:10.1186/s12711-016-0250-9) contains supplementary material, which is available to authorized users.

## Background

Genomic selection and genomic breeding value estimation were implemented in several cattle breeding programs in the last few years. Since the introduction of this methodology, there has been a constant attempt to further improve it and to increase the reliabilities of genomic breeding values. One key factor is the size of the reference set [1, 2]. Nowadays, there are several international organizations that promote the exchange of genotypes on a regular basis to enlarge reference sets and to improve the quality of genomic predictions of the participating countries. In dual-purpose Fleckvieh (FV) cattle, genomic selection was implemented in 2011 and genetic evaluation centers in Germany and Austria cooperate in a joint genetic and genomic evaluation that uses a common genotype pool [3]. Currently, the reference set for FV includes approximately 9000 bulls with phenotypic measures on most traits.

Several studies have reported that sharing genotypes within breeds results in large benefits for the reliability of genomic predictions e.g. [4–6]. However, most opportunities to increase the genotype pool by exchanging genotypes have been exploited and, in most cases, the growth of reference sets within breeds is restricted to the yearly increase in number of genomically preselected young bulls receiving daughter proofs. As a consequence, fewer bulls are progeny-tested than in pre-genomic selection programs [7, 8] and the proportion of old bulls increases over time. Since the reliability of genomic predictions also depends on the degree of relationship between reference and predicted animals [9], this ‘aging’ of the reference set may lead to decreased reliabilities. As a demonstration of that effect, Cooper et al. [10], for example, excluded subsets of old bulls and found that older bulls in the reference set had only a minimal impact on the reliability of the genomic breeding values of predicted animals. In addition, preselection of young reference bulls may influence the quality of genomic predictions. Schaeffer [11] predicted a situation where considerable bias was introduced on genomic evaluations by strong preselection [12–14] of young bulls based on their genomic breeding values.

Another possibility to increase the size of the reference set is to use information from genotyped and phenotyped females, which can have a beneficial influence on the quality of genomic predictions. Thomasen et al. [15] found that, by adding female genotypes in the reference set, more genetic gain with a lower rate of inbreeding can be achieved compared to a breeding scheme where the reference set grows only from the addition of newly progeny-tested bulls. Pryce et al. [8] showed that by adding 10,000 cows to a reference set of 3000 Holstein bulls, the reliability of genomic predictions of 437 young bulls in the validation set was improved by 4 to 8 %. Calus et al. [16] also combined cows and bulls in a single reference set and found that the highest validation accuracies were achieved with the combined dataset compared to scenarios with a reference set that included only cows or only bulls. Furthermore, since usually cows are not strongly preselected, inclusion of their genotypes and phenotypes may also contribute to reduce the biasing effects of preselection as pointed out by Schaeffer [11]. Last but not least, genotyping cows might be especially important for creating reference sets for so-called new traits or expensive-to-measure traits [7, 17, 18] and, most likely, will be the basis of new and useful management tools for farmers [8].

If female genotypes are to be included in a genomic system, one of the key questions is which cows should be genotyped. Pryce et al. [8], Wiggans et al. [19] and Dassonneville et al. [20] discussed preferential treatment as a potential problem related to the inclusion of bulls’ dams into the reference set. Dassonneville et al. [20] found that the inclusion of records on elite cows resulted in overestimation of genomic enhanced breeding values for all animals. Thus, even if genotypes are available for elite cows as a consequence of using genomic predictions for the selection of bulls’ dams, in the end, they should not be part of the reference set.

In a preliminary study [21], we performed a deterministic simulation based on nuclear pedigrees extracted from the German-Austrian FV population and showed that there is a benefit from including genotyped cows into the reference set. We quantified the effects of this inclusion on the reliability of genomic breeding values of young selection candidates and found marginal to considerable gains in reliability (between 1 and 40 %) depending on the scenario. However, we were not able to quantify the effects of selection on the results and we could not quantify the cumulative effects at the population level. Therefore, in this study, we examined the following three main effects by means of a stochastic simulation: (1) effects of selection on validation reliability, (2) effects of genotyping randomly selected cows on the accuracy of prediction, and (3) effects of some alternative strategies for sampling the genotyped daughters.

## Methods

### Simulation

We used the open access software QMSim [22] to run a simulation with five repetitions. Our aim was to simulate a population that resembled the German-Austrian dual-purpose Fleckvieh cattle population for several key characteristics (e.g. linkage disequilibrium (LD) structure, allele frequencies and effective population size).

#### Simulation of the population

QMSim first simulated a so-called historical population, which consisted of 2000 unrelated animals with a balanced sex ratio. These animals were randomly mated for 2500 generations. To create a sufficiently strong LD structure as observed in FV, a bottleneck was introduced after 2500 generations by reducing the number of breeding animals to 150 for one generation, which corresponds approximately to the effective population size in FV i.e. 160 based on the observed LD structure [23]. This estimate is quite close to that based on pedigree data [24]. After this bottleneck, population size was increased within one generation again to 31,500 animals (30,000 dams and 1500 sires), which represented the founder animals (generation 0) of the so-called ‘recent’ or pedigreed population. The recent population was propagated for another 10 generations. In each generation of the recent population, 15,000 female and 15,000 male offspring were generated by mating 30,000 dams and 1500 breeding sires. Generations overlapped and in each generation 30 % of the dams and 70 % of the sires were replaced. These two replacement parameters were quite similar to the situation observed in the real FV population. Breeding animals were selected based on their estimated breeding value (EBV) which was calculated within QMSim with a reliability of 0.6. This was done to mimic a genomic selection program where dams are selected based on a combination of pedigree information and own performance and sires are selected on their genomic breeding value.

**Fig. 1:**
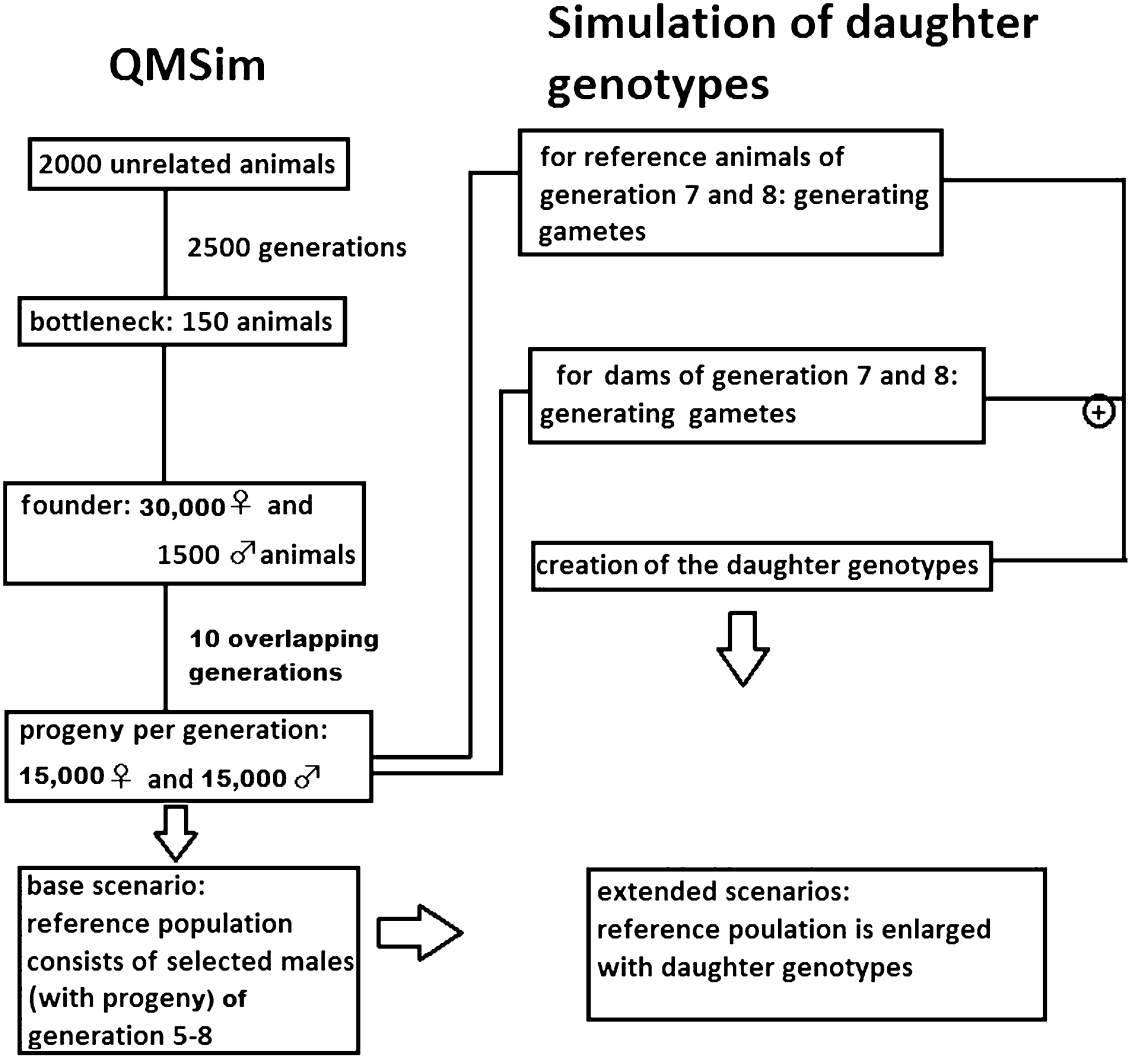
Structure of the simulation

Males of generation 5 to 10 were genotyped (Table 1). Sires belonging to generations 5 to 8 (n = 4200) were assigned to the reference set. The remaining animals of generations 9 and 10 were used as validation set for forward prediction. Note that whereas sires in generation 9 (n = 1050) were young bulls that were selected by QMSim based on a genomic breeding value but without daughter performances, the animals of generation 10 (n = 15,000) were unselected candidates. The validation animals were further characterized by the status of their sire i.e. a reference animal or not. Figure 1 gives an overview of the structure of the simulation.

**Table 1:** Assignment of animals to the reference or validation set in the different scenarios

Generation	Number of individuals			Explanation
	Base scenario	Extended scenarios step 1	Extended scenarios step 2	
5	1050	1050	1050	Reference set
6	1050	1050	1050	
7	1050	1050	1050 + daughters	
8	1050	1050 + daughters	1050 + daughters	
9	1050	1050	1050	Validation set
10a	4516	4516	4516	
10b	10,484	10,484	10,484	

Validation animals were further divided according to the status of the corresponding sire (member of the reference set or not), resulting in three validation groups. Sires of validation animals in generations 9 and 10a were part of the reference set and sires of validation animals in generation 10b were not part of the reference set. First, daughters of the sires of generation 8 were added to the reference set (step 1) and then daughters of the sires of generation 7 were also added (step 2)

#### Simulation of the genome

We simulated 30 chromosomes, each 100 cM long. On each chromosome, 1660 single nucleotide polymorphisms (SNPs) and 30 quantitative trait loci (QTL) were evenly distributed (49,800 SNPs and 900 QTL in total). After routine checks [3, 25], nearly 38,000 valid SNPs and approximately 700 QTL that were still segregating in the reference set (both numbers slightly varying between replicates of the simulation) were available. The routine checks were as follows: (1) SNPs that deviated from Hardy–Weinberg equilibrium (HWE) with a p-value less than 10^−5^ and (2) SNPs with a minor allele frequency (MAF) lower than 0.02 were excluded from the dataset. We assumed a sex-linked trait and a single observation for each female with a heritability set to 0.4. The polygenic nature of the trait was ensured by the relatively large number of QTL and their effects were drawn from a uniform distribution (option ‘uniform’ from QMSim) to prevent the occurrence of a few isolated large QTL effects. With a uniform distribution, the mean of the effects is related to the variance and, thus, the range of the QTL effects is limited. We performed a couple of tests with QMSim and the results confirmed our assumptions (data not shown).

#### Simulation of the daughter sets

In the main part of the simulation, we generated 200 daughters for each of the reference bulls of generations 7 and 8 (which represented a total of 420,000 additional female genotypes and phenotypes). Due to memory requirements and some limitations of the QMSim software, we did not simulate the daughter genotypes with QMSim directly. Instead, based on the known haplotypes (SNPs and QTL) of the reference bulls of these two generations, we simulated different male gametes by recombination and randomly mated them with gametes of potential dams of the same cohort (excluding sisters, daughters and dams) that was simulated by applying the same strategy. Assuming a Poisson distribution for cross-overs, recombination was simulated by generating on average one random cross-over per Morgan for each chromosome. Using the observed QTL status of each daughter and the known (true) QTL effects from the QMSim simulation, we calculated the true breeding value (TBV) for each daughter.

### Phenotypes

We generated yield deviations (YD, [26]) for daughters using the TBV and a random residual. Depending on the design investigated, these daughter phenotypes were used to calculate daughter yield deviations (DYD, [26]) of the corresponding bull or were directly included in the reference set. In this way, YD of the reference daughters were automatically omitted from the daughter yield deviation (DYD) of the sire and double-counting in the extended scenarios was avoided. To account for different variances of the YD and DYD, phenotypes were weighted with the equivalent number of own performances (EOP, [27]) calculated as

$${\text{EOP}} = \lambda \frac{{{\text{R}}_{\text{phen}}^{2} }}{{1 {-} R_{\text{phen}}^{2} }},$$

where $\uplambda = \frac{{\upsigma_{\text{e}}^{2} }}{{\upsigma_{\text{a}}^{2} }}$ with $\upsigma_{\text{a}}^{2}$ being the additive genetic variance and $\upsigma_{\text{e}}^{2}$ the residual variance and ${\text{R}}_{\text{phen}}^{2}$ the reliability of the DYD or YD.

### Designs

In a more general analysis, we investigated the effects of selection on validation reliability and model-derived reliability parameters. To be able to identify these selection effects, we repeated the basic scenario using the same parameters for QMSim except that we replaced directional selection on EBV by random selection.

In the main part of the simulation, we included large numbers of genotyped cows into the reference set. The general sampling strategy was to genotype a random sample of fixed size of phenotyped daughters of each artificial insemination (AI) bull in defined cohorts. We investigated 10 different scenarios: one base scenario and nine extended scenarios. In the base scenario, the reference set consisted only of sires of generations 5 to 8. For the extended scenarios, an increasing number of the generated female genotypes and phenotypes were integrated into the reference set. Tables 1 and 2 give an overview of the different scenarios.

**Table 2:** Scenarios with corresponding number of animals and composition of the reference set

Scenario	Reference set	
	Number of sires	Number of daughters
Base	4200	0
–/25	4200	26,250
–/50	4200	52,500
–/100	4200	105,000
–/200	4200	210,000
50/50	4200	105,000
100/100	4200	210,000
200/200	4200	420,000
–/50_s_	4200	52,500
–/25_r_25_s_	4200	52,500
–/50_ub_	4200	52,500

The names of the extended scenarios are derived from the number of daughters per sire which are included in the reference set and the sire’s generation. The number before the slash in the scenario’s name is the number of daughters per progeny-tested bull of generation 7 (i.e. step 2 of the extended scenarios) and the number after the slash is the number of daughters per progeny-tested bull of generation 8 (i.e. step 1 of the extended scenarios). The –/50_s_ is a scenario in which the best daughters were selected to be genotyped, –/25_r_25_s_ is a scenario in which 25 random daughters per sire and the 25 best daughters per sire were selected and genotyped and –/50_ub_ is a scenario in which an unbalanced number of daughters for all sires was selected

To assess how robust the benefits are with respect to our general sampling strategy, we changed the composition of the sample of scenario –/50 (Table 2). Instead of including a random sample of daughters as was done in scenario –/50, we selected the best 50 daughters of each sire for scenario –/50_s_ (selection was done on YD). In the scenario –/25_r_25_s_, we selected 25 daughters at random and combined them with the 25 best remaining daughters of the corresponding sire. Finally, we also ran one unbalanced scenario (–/50_ub_) with different numbers of daughters per sire to test the effect of moderate unbalancedness but the overall number of genotyped females was kept the same as in scenario –/50. This was done by randomly selecting five daughters for 330 sires, 50 daughters for 621 sires and all 200 daughters for 99 sires. The different numbers of the daughter sets per sire were chosen arbitrarily but we ensured that the total number of genotyped females was maintained and that each sire was represented by at least some daughters. Moreover, random assignment of the different numbers of daughters to the sires was also conducted.

### Genomic prediction

Due to the large number of genotypes, we used a SNP-best linear unbiased prediction (BLUP) model [28] to calculate direct genomic values (DGV) and reliabilities. The model equation is as follows:

$${\mathbf{y}} = {\mathbf{Xb}} + {\mathbf{Mg}} + {\mathbf{e}},$$

and the corresponding mixed model equations are:

$$\left( {\begin{array}{*{20}l} {{\mathbf{X}}^{{\prime }} {\mathbf{R}}^{{ - {\mathbf{1}}}} {\mathbf{X}}} \hfill &\quad {{\mathbf{X}}^{{\prime }} {\mathbf{R}}^{{ - {\mathbf{1}}}} {\mathbf{M}}} \hfill \\ {{\mathbf{M}}^{{\prime }} {\mathbf{R}}^{{ - {\mathbf{1}}}} {\mathbf{X}}} \hfill &\quad {{\mathbf{M}}^{{\prime }} {\mathbf{R}}^{ - 1} {\mathbf{M}} + {\mathbf{I}}/\sigma_{g}^{2} } \hfill \\ \end{array} } \right)\left( {\begin{array}{*{20}c} {\hat{\mathbf{b}}} \\ {\hat{\mathbf{g}}} \\ \end{array} } \right) = \left( {\begin{array}{*{20}c} {{\mathbf{X}}^{{\prime }} {\mathbf{R}}^{{ - {\mathbf{1}}}} {\mathbf{y}}} \\ {{\mathbf{M}}^{{\prime }} {\mathbf{R}}^{{ - {\mathbf{1}}}} {\mathbf{y}}} \\ \end{array} } \right)$$

where

$$\upsigma_{\text{g}}^{2} = \frac{{\upsigma_{\text{a}}^{2} }}{{\sum_{{{\text{j}} = 1}}^{\text{m}} (2{\text{p}}_{\text{j}} {\text{q}}_{\text{j}} )}},$$

and ${\mathbf{y}}$ is the vector of observations (here DYD or YD), ${\mathbf{b}}$ the vector of fixed effects (in our simulation only an overall mean), ${\mathbf{g}}$ is the vector of random marker effects, and ${\mathbf{e}}$ the vector of residual effects. Matrix ${\mathbf{X}}$ is a design matrix which links the observations to the respective fixed effects and ${\mathbf{M}}$ is the centered coefficient matrix of marker genotypes and ${\text{p}}_{\text{j}}$ and ${\text{q}}_{\text{j}}$ are base allele frequencies of marker j estimated for generation 0 [29]. Centering was done by subtracting two times the base allele frequency estimate from the corresponding column of ${\mathbf{M}}$. Matrix ${\mathbf{R}}$ is a diagonal matrix with $\upsigma_{\text{e}}^{2} /{\text{w}}_{\text{i}}$ on the diagonal, where ${\text{w}}_{\text{i}}$ is the EOP of the ${\text{i}}$-th observation and matrix ${\mathbf{I}}$ is an identity matrix of order $m$ (number of markers).

${\text{DGV}}$ are calculated as:

$${\text{DGV}} = {\hat{\mathbf{b}}} + {\mathbf{M}}{\hat{\mathbf{g}}},$$

and the corresponding predicted error variances (${\text{pev}}$) are calculated as:

$${\text{pev}}\left( {\text{DGV}} \right) = {\mathbf{M}}^{*} {\mathbf{C}}_{\text{s}}^{ - 1} {\mathbf{M}}^{{*{\prime }}} ,$$

where ${\mathbf{M}}^{*}$ is matrix ${\mathbf{M}}$ extended with a column of ones, and ${\mathbf{C}}_{\text{s}}^{ - 1}$ is the inverse of the left hand side of the SNP-BLUP-MME (mixed model equation). The inclusion of the overall mean in the calculation of the pev can be questioned and may lead to slightly higher theoretical reliabilities. We empirically compared results including and omitting the overall mean and found differences that were smaller than the rounding precision of the results. Moreover, because the overall mean is included in each scenario, its impact on the contrasts between scenarios can be ignored.

The reliability of the DGV of the ${\text{i}}$-th animal can then be calculated as:

$${\text{R}}_{\text{i}}^{2} = 1 - \frac{{{\text{diag}}({\text{pev}}({\text{DGV}}))_{i} }}{{{\text{diag}}({\mathbf{G}})_{\text{i}}\upsigma_{\text{a}}^{2} }},$$

where ${\text{diag}}({\text{pev}}({\text{DGV}}))_{\text{i}}$ is the i-th diagonal element of the ${\text{pev}}\left( {\text{DGV}} \right)$ and ${\text{diag}}({\mathbf{G}})_{\text{i}}$ the ${\text{i}}$-th diagonal element of the genomic relationship matrix (${\mathbf{G}}$) which is 1 plus the genomic inbreeding coefficient. Matrix ${\mathbf{G}}$ is defined as follows:

$${\mathbf{G}} = \frac{{{\mathbf{MM}}^{{\prime }} }}{{\sum_{{{\text{j}} = 1}}^{\text{m}} (2{\text{p}}_{\text{j}} {\text{q}}_{\text{j}} )}}.$$

In addition, we calculated a weighted regression of TBV on ${\text{DGV}}$ for validation animals. We used the model fit of this regression as a measure of validation reliability ($\uprho^{2}$) and the slope (b) as a measure of the bias that describes the inflation of estimates [30].

To quantify the effect of incomplete LD between SNPs and QTL on the difference between model-derived theoretical reliabilities and validation reliabilities, we included an analysis where we extended the marker genotype coefficient matrix ${\mathbf{M}}$ by QTL genotypes. We present the results in the context of the comparison between designs with directional and with random selection ($\uprho_{\text{QTL}}^{2}$).

## Results

### Comparison of the simulated dataset with the Fleckvieh population

Comparison of the extent of LD between the simulated dataset and the real Fleckvieh dataset [31], revealed a good agreement with slightly higher values of the linkage parameter ${\text{r}}^{2}$ [32] for the simulated data at shorter distances. The average distance between a QTL and the nearest SNP in the simulated data was 60 kb. Allele frequencies for the simulated dataset were more evenly distributed than those for the real FV data, for which a slight shift to lower allele frequencies was observed. These results are illustrated in Figure S1 [see Additional file 1: Figure S1] and Figure S2 [see Additional file 2: Figure S2].

### Simulation

For ease of interpretation, we separated the presentation of results for generation 9 from those for generation 10, in order to highlight the fact that generation 9 represents a group of individuals that are already pre-selected on an EBV including Mendelian sampling information in the course of the simulation process. This selection does have an effect on validation statistics [30]. In contrast, generation 10 is strictly unselected. Results for generation 10 were further divided according to the status of the sire (member of the reference group or not). A more detailed categorization of the results for these two generations is provided in Tables S1 and S2 [see Additional file 3: Tables S1 and S2]. There was a general tendency for scenarios with the same number of genotyped females (scenario –/100 compared to scenario 50/50 and scenario –/200 compared to scenario 100/100) showing nearly identical results. For the sake of clarity, we do not present results for the redundant scenarios. All the results shown are averages over five repetitions of the simulation. Standard errors of the results presented in the main body of the paper were less than 1.3 % for validation reliabilities (except for one scenario i.e. –/25_r_25_s_ where standard errors were between 3.2 and 4.1 %) and less than 0.02 for regression slopes.

### General effects of selection

Table 3 shows model-derived reliabilities (${\text{R}}^{2}$) and validation reliabilities ($\uprho^{2}$) for a scenario with directional selection and a scenario with random selection. Model-derived reliabilities were slightly higher for the scenario with directional selection than for the scenario with random selection, which indicated that, with directional selection, the pattern of family sizes differs and results in a more informative structure for validation animals. Comparing ${\text{R}}^{2}$ with $\uprho^{2}$ for randomly selected populations, we found slightly lower validation reliabilities when only SNPs were considered. When QTL were included in the SNP panel, validation reliabilities ($\uprho_{\text{QTL}}^{2}$) were slightly higher than ${\text{R}}^{2}$. In the scenario with directional selection the validation reliabilities for generation 10 were lower than with random selection (40 to 51 and 33 to 40 %, respectively). When, in addition, the validation sample was selected on information that included Mendelian sampling information as in generation 9, the decrease in validation reliabilities was even more pronounced (26 to 51 %). Selection on parent average (PA) in the validation group did not result in inflated predictions (slope estimates ranged from 0.93 to 0.99 for generation 10a).

**Table 3:** Model-derived reliabilities (${\mathbf{R}}^{2}$) and validation reliabilities (${\varvec{\uprho}}^{2}$) in the base scenario with directional and random selection

Validation set	Sire status	Number of individuals	Base scenario					
			Random selection			Directional selection		
			${\mathbf{R}}^{2}$	${\varvec{\uprho}}^{2}$	${\varvec{\uprho}}_{{{\mathbf{QTL}}}}^{2}$	${\mathbf{R}}^{2}$	${\varvec{\uprho}}^{2}$	${\varvec{\uprho}}_{{{\mathbf{QTL}}}}^{2}$
9	Reference	1050	54	51	59	58	26	32
10a	Reference	4516	54	51	58	58	40	48
10b	Not reference	10,484	48	40	49	48	33	41

Validation animals were divided according to whether their sire was in the reference set or not. For the purpose of illustration (and only here), we included results of analyses in which the segregating QTL were included in the SNP panel used for estimation and prediction (${\varvec{\uprho}}_{{{\mathbf{QTL}}}}^{2}$)

### Effects of genotyped daughters

Table 4 presents validation reliabilities for the three validation groups for the basic scenario and five extended scenarios. Using results for group 10a as a starting point, it can generally be stated that introducing an increasing number of genotyped daughters into the reference set clearly had a positive impact on the validation reliability. Beginning with scenario –/100, validation reliabilities reached values of 70 % and more. If the sire of a validation animal was not a member of the reference set (generation 10b), the overall validation reliability was reduced, but the general trend observed was the same. As expected, the effect of the contribution of a missing sire to the overall reliability decreased as information increased. When the validation group itself was selected (generation 9), the validation reliabilities for all scenarios were lower than for the other validation groups. Again, the impact of this decrease was more pronounced when the number of cows in the reference set was smaller.

**Table 4:** Validation reliability (${\varvec{\uprho}}^{2}$) for six different scenarios

Validation set	Sire status	Number of individuals	${\varvec{\uprho}}^{2}$					
			Base	–/25	–/50	–/100	100/100	200/200
9	Reference	1050	26	44	53	62	72	80
10a	Reference	4516	40	56	65	73	80	86
10b	Not reference	10,484	32	51	60	69	77	84

Validation animals were divided according to whether their sire was in the reference set or not

### Effects of the composition of the daughter samples

Table 5 illustrates some aspects of the composition of the sample of daughters that were chosen for genotyping. Starting with values for ${\text{R}}^{2}$, $\uprho^{2}$ and b for scenario –/50 as a reference point, we found a lower validation reliability and a noticeable increase in inflation of genomic predictions when a selected daughter group was genotyped (scenario –/50_s_), even if selection was based on the criterion of moderate reliability as in this case. Comparing the base scenario (Table 4) to scenario –/50_s_ (Table 5), the benefit from adding 52,500 genotyped daughters was small with respect to validation reliability. The negative effect of this preselection can be partially compensated by a combination of directly and randomly selected daughters (scenario –/25_r_25_s_, Table 5), but nevertheless the results were lower than those for a scenario where only 25 randomly selected daughters per sire were included (scenario –/25, see Table 4). A moderately unbalanced scenario (scenario –/50_ub_, Table 5), however, had no detectable effect on reliabilities or regression slopes.

**Table 5:** Model-derived reliabilities (${\mathbf{R}}^{2}$ were virtually equal across all scenarios), validation reliability (${\varvec{\uprho}}^{2}$) and regression slopes of the –/50 scenario and the three additional scenarios

Scenarios				–/50		–/50_s_		–/25_r_25_s_^a^		–/50_ub_	
Validation set	Sire status	Number of individuals	${\mathbf{R}}^{2}$	${\varvec{\uprho}}^{2}$	b	${\varvec{\uprho}}^{2}$	b	${\varvec{\uprho}}^{2}$	b	${\varvec{\uprho}}^{2}$	b
9	Reference	1050	81	53	0.82	35	0.60	40	0.98	53	0.79
10a	Reference	4516	81	65	0.95	42	0.76	48	1.22	65	0.95
10b	Not reference	10,484	76	60	0.92	37	0.70	44	1.14	60	0.91

Validation animals were divided according to whether their sire was in the reference set or not

^a^Higher standard error compared to the other scenarios

## Discussion

In this study, we show that even small groups of daughters per sire can have large beneficial effects on model-derived reliabilities as well as validation reliabilities. A straightforward strategy to achieve these beneficial effects is to genotype a balanced random sample of daughters per sire. With respect to the structure of the validation sample, the results for generation 10 represent the ideal validation sample because it comprises the complete male offspring of the previous generation. In the following discussion, we refer to the results for validation group 10a unless otherwise indicated.

### Effects of selection

This section is included to illustrate the general effects of selection on validation statistics and to clarify the extent to which the results obtained can be explained by the fact that our population is under selection. The results (Table 3) are in good agreement with expectations and results found by other authors [33–35]. Surprisingly, at first, model-derived theoretical reliabilities were slightly higher for the scenario with directional selection than for the scenario with random selection. However, by analyzing family structures, we found that with directional selection the pattern of family sizes differed, resulting in a more informative structure for validation animals in scenarios with directional selection (results not shown). Model-derived theoretical reliabilities and validation reliabilities show relatively good agreement for the scenario with random selection. The slightly lower values for validation reliabilities are presumably a consequence of the fact that the LD between SNPs and QTL is not perfect and consequently some parts of the additive-genetic variance are not captured by SNPs [36]. However, by simply adding the QTL to the model, we found that validation reliabilities were slightly higher than model-derived theoretical reliabilities. In this case also, the theoretical model is only an approximation of the underlying true model.

The lower values for validation reliabilities under directional selection must be considered as a consequence of selection in the parental generation [33]. When the validation sample itself was selected on a criterion that included Mendelian sampling information, as was the case in generation 9, the decrease in validation reliabilities was even more pronounced. These results are in agreement with previous studies about the effects of selection on theoretical and validation reliabilities [35, 37].

### Size and structure of the daughter samples

We tested different scenarios for which increasing numbers of genotyped and phenotyped daughters per sire were included in the reference set. By genotyping 25 daughters per sire from a single generation (corresponding to an overall number of 26,250 genotyped females, Table 2), the validation reliability was considerably improved, from 40 % in the base scenario to 56 % (Table 4, scenario –/25). As the number of daughters increased, the validation reliability showed a nearly linear increase. If we assume that proofs from progeny-testing typically show a validation reliability of about 70 % [38], this threshold is reached in scenario –/100 for validation group 10a and in scenario 100/100 for all other validation groups. With the largest number of genotyped daughters in scenario 200/200 (corresponding to a total of 420,000 genotyped females in the reference set), all validation groups reached reliabilities of 80 % or more. This indicates that large numbers of (unselected) females in the reference set can largely compensate for unfavorable effects such as selection in the parental generation or the effect of a sire for which daughter proofs are not available. As already mentioned, we did not find any relevant differences between scenarios with equal total numbers of females (e.g. scenarios 50/50 and –/100). The similarity between the results of these scenarios is interesting. We expected that a scenario with daughters from two generations such as scenario 50/50 would lead to (slightly) higher validation reliabilities than scenario –/100 because with overlapping generations a larger number of sires would have genotyped daughters in scenario 50/50 and therefore more haplotypes would have been sampled. However, it seems that the existing diversity of haplotypes is already sufficiently covered when genotyping only one generation. In addition, beneficial effects can be reduced by an additional round of meiosis [21]. This implies that a large fraction of the benefits can be already generated in the first generation of a genotyping strategy that considers randomly selected females. Other studies found increases in validation reliabilities when including cows in the reference set but the reported increases were generally much lower e.g. [8, 16, 39]. We see several reasons for such differences. The most obvious one is certainly the larger number of cows that were assumed to be genotyped and phenotyped. Pryce et al. [8] and Koivula et al. [39] added approximately 10,000 genotyped cows to the reference set and Calus et al. [16] only ~1600 first lactation heifers. Other reasons might be related to key parameters such as the reliability of the phenotype [36], effective population size or the LD structure. Moreover, all studies mentioned above used real data that can be differently influenced by selection.

The concept that we propose here is based on genotyping and phenotyping a random sample of (preferably) first-crop daughters of each sire from a generation. We examined how deviations from this design would influence results. Comparison of the results of scenario –/50 (random daughter sample, Table 4) and scenario –/50_s_ (selected daughter sample, Table 5), showed that with scenario –/50_s_ the beneficial effect of an additional pool of 52,500 genotypes in the reference set on validation reliability is almost null when compared to the base scenario. Even worse, preselection of daughters caused an increase in inflation as indicated by the low regression slopes (Table 5). One possible explanation is that reference animals that are selected based on their within-family deviation lead to biased family means and also to biased estimates of the deviations from the family mean. Schaeffer [11] argued that the animal model might become obsolete due to the fact that, in the future, only preselected young bulls will become reference animals. The consequence of this preselection would be that the phenotyped sons of a sire would not represent a random sample of all sons of this sire. Schaeffer [11] expected a relevant increase in inflation as a consequence of this development and given our results this expectation might be at least partly justified. Although not explicitly covered here, it seems likely that the integration of elite cows in the reference set will result in an even stronger bias, because elite cows are not only selected, they frequently receive also preferential treatment so that even their phenotypes are biased. Studies of Wiggans et al. [19] and Dassonneville et al. [20] dealt with the consequences of preferential treatment and provide further evidence of its biasing effects.

The negative result of scenario –/50_s_ can be only partly removed by a combination of selected and unselected daughters (scenario –/25_r_25_s_; same number of daughters, Table 5). This result indicates that the combination of selected and unselected data cannot yield precise and unbiased estimates. Moreover, the results of scenario –/25_r_25_s_ are lower than those of scenario –/25 (Table 4), which indicates that it might be relevant to exclude the genotypes of (pre-)selected daughters from the reference set if this information is available. This kind of monitoring presents an additional challenge especially to single-step genomic BLUP, in which putting a restriction on the reference set is not conceptually intended, an important aspect that was already emphasized by other authors [40].

Another factor with a strong impact on the validation results is the heritability of the trait. In a pilot study [21], we found that for traits with medium to high heritabilities (h^2^ = 0.35), 100 genotyped daughters per bull increased the marginal reliability [41] by up to 17 % (depending on the scenario) whereas in situations with very low heritabilities (h^2^ = 0.05), the same number of daughters increased the reliability by up to 4 % only. Our study was limited to a trait with a heritability of 0.4 to investigate several other questions. However, it may be expected that with a lower heritability, less substantial improvements would be found.

In the literature, there are other strategies for genotyping cows. Jiménez-Montero et al. [42] found higher reliabilities when cows selected from both extremes of the distribution of phenotypes were genotyped instead of the best ones or a random sample. We hypothesize that such a strategy would be better suited for traits for which only a few QTL with large effects segregate. Such traits are not common in dairy cattle [43] and therefore we focused our study on a trait with polygenic characteristics, for which no advantage of genotyping extreme animals is expected. Moreover, such a sampling strategy would require trait-specific daughter samples, which is an obstacle for practical implementation. In Calus et al. [16], cow genotypes of entire herds are integrated in the reference set. This strategy could indeed ensure the representativeness of the cow sample if some precautions are taken. We found no disadvantages with moderate unbalancedness in scenario –/50_ub_ in which we ensured that each bull was at least represented by a sample of five daughters. Further investigations on this subject are necessary to clarify which degree of unbalancedness can be tolerated before the accuracy of prediction deteriorates.

In real world breeding programs, it is reasonable to assume that there is a limited interest for the farmers to genotype randomly selected cows and to keep all of them for an unbiased performance recording. Thus, for practical implementation, it would be necessary to find a solution to finance the genotyping costs and to keep track of the cows sampled for the reference set. However, this independent financing solution, once established as a component of the breeding program, might be the only way to ensure a neutral, unselected daughter sample in the long term.

The simple balanced genotyping designs proposed here led to very stable improvements as indicated by the small standard errors of reliabilities and slopes. The only exception was for scenario –/25_r_25_s_, which showed more variation in the results. This indicates that some sampling designs are more robust than others with respect to the improvements that can be achieved.

## Conclusions

Extending the reference set by adding a large number of cows with genotypes and phenotypes increases the reliability of breeding values of young selection candidates and may overcome the deterioration of validation reliabilities that are caused by intense preselection of young bulls. We showed the benefits from genotyping a random sample of (first-crop) daughters of all sires from one or two generations. It is possible to obtain reliabilities for selection candidates that are as high as, or even higher than, the reliabilities that have been formerly observed for young progeny-tested bulls. We found that the benefits that can be achieved are sensitive to the strategy used to sample females for genotyping.

## Additional files

10.1186/s12711-016-0250-9 LD-structure of the real Fleckvieh population (r2_Fleckvieh data, [30]) and of the simulated population (r2_simulated data) according to distance between SNPs in kb.
10.1186/s12711-016-0250-9 Distribution of the allele frequencies. (A) Simulated data, approximately 38,000 segregating SNPs; (B) Real data on Fleckvieh cattle, approximately 41,000 segregating SNPs.
10.1186/s12711-016-0250-9 More detailed results on validation reliabilities (ρ^2^) (**Table S1**) and on the model-derived theoretical reliabilities (R^2^) (**Table S2**) for six scenarios. Description: Validation animals were divided according to the generation of their sire.
