# Supplementary figures and images for: Systematic genotyping of groups of cows to improve genomic estimated breeding values of selection candidates
Source: Genet Sel Evol. 2016 Sep 28;48:73. doi: 10.1186/s12711-016-0250-9 (PMC5039940; doi:10.1186/s12711-016-0250-9)

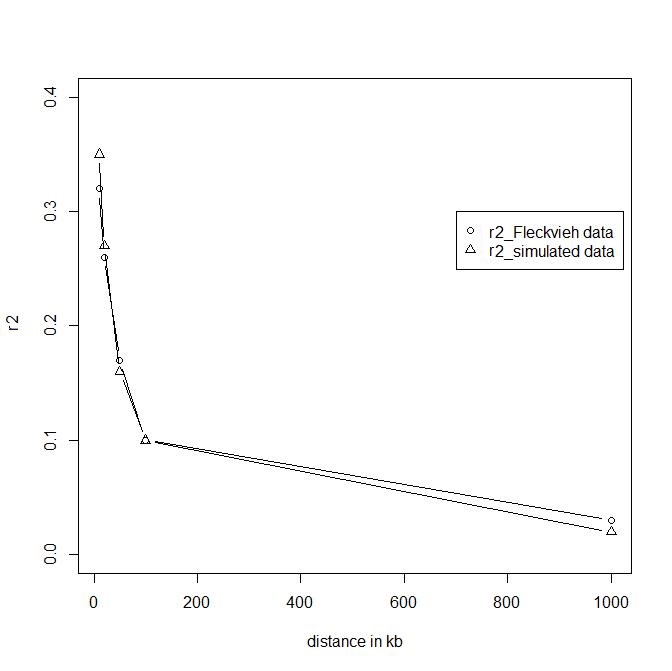

Supplement: Supplementary file 1 — 10.1186/s12711-016-0250-9 LD-structure of the real Fleckvieh population (r2_Fleckvieh data, [30]) and of the simulated population (r2_simulated data) according to distance between SNPs in kb. [file 12711_2016_250_MOESM1_ESM.jpg]

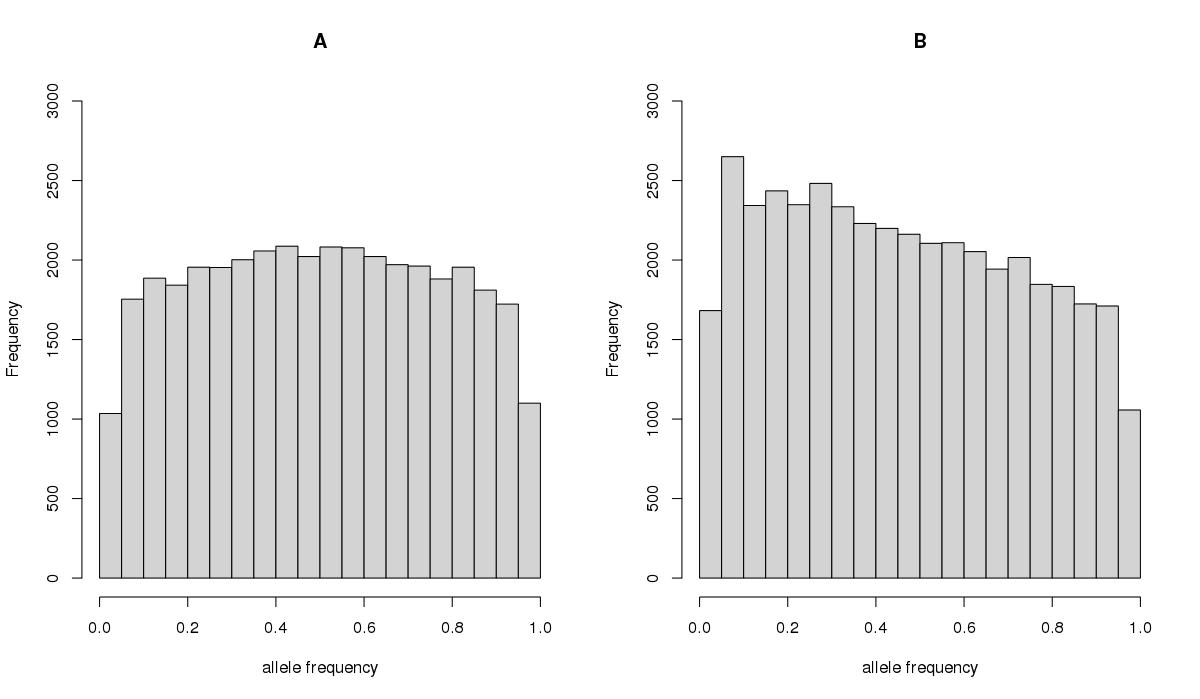

Supplement: Supplementary file 2 — 10.1186/s12711-016-0250-9 Distribution of the allele frequencies. (A) Simulated data, approximately 38,000 segregating SNPs; (B) Real data on Fleckvieh cattle, approximately 41,000 segregating SNPs. [file 12711_2016_250_MOESM2_ESM.jpg]
