# Supplementary material for: Systematic genotyping of groups of cows to improve genomic estimated breeding values of selection candidates
Source: Genet Sel Evol. 2016 Sep 28;48:73. doi: 10.1186/s12711-016-0250-9 (PMC5039940; doi:10.1186/s12711-016-0250-9)
Supplement: Supplementary file 3 — 10.1186/s12711-016-0250-9 More detailed results on validation reliabilities (ρ2) (Table S1) and on the model-derived theoretical reliabilities (R2) (Table S2) for six scenarios. Description: Validation animals were divided according to the generation of their sire. [file 12711_2016_250_MOESM3_ESM.docx]

Additional file 3 Tables S1 and S2

Table S1

| **Validation generation** | **Sire generation** | **Number** | **ρ^2^** | | | | | |
| --- | --- | --- | --- | --- | --- | --- | --- | --- |
|  |  |  | **Base** | **--/25** | **--/50** | **--/100** | **100/100** | **200/200** |
| 9 | 8 | 731 | 26 | 44 | 53 | 62 | 72 | 80 |
|  | 7 | 228 | 26 | 44 | 55 | 63 | 74 | 80 |
|  | 5/6 | 86 | 24 | 42 | 51 | 61 | 69 | 77 |
| 10 | 9 | 10,484 | 32 | 51 | 60 | 69 | 77 | 84 |
|  | 8 | 3086 | 41 | 57 | 65 | 73 | 80 | 86 |
|  | 7 | 1099 | 40 | 55 | 65 | 72 | 80 | 85 |
|  | 5/6 | 310 | 37 | 56 | 64 | 73 | 81 | 86 |

Table S2

| **Validation generation** | **Sire generation** | **Number** | **R^2^** | | | | | |
| --- | --- | --- | --- | --- | --- | --- | --- | --- |
|  |  |  | **Base** | **--/25** | **--/50** | **--/100** | **100/100** | **200/200** |
| 9 | 8 | 731 | 58 | 75 | 81 | 87 | 91 | 94 |
|  | 7 | 228 | 58 | 75 | 81 | 87 | 91 | 95 |
|  | 5/6 | 86 | 58 | 75 | 81 | 87 | 91 | 95 |
| 10 | 9 | 10,484 | 48 | 69 | 76 | 83 | 89 | 93 |
|  | 8 | 3086 | 57 | 74 | 81 | 86 | 91 | 94 |
|  | 7 | 1099 | 58 | 75 | 81 | 86 | 91 | 94 |
|  | 5/6 | 310 | 58 | 75 | 81 | 87 | 91 | 95 |
